# Predicting coronary artery lesions in patients with Kawasaki disease in China using a machine-learning algorithm: a retrospective cohort study

**DOI:** 10.3389/fimmu.2026.1807028

**Published:** 2026-04-22

**Authors:** Xuemei Li, Zihan Zhou, Jingyi Fan, Lin Zhao, Ruidi Xu, Dong Li, Xu Ma, Lu Sun, Yujian Wu, Zhouping Wang, Ce Wang

**Affiliations:** 1 Department of Pediatrics, Shengjing Hospital of China Medical University, Shenyang, Liaoning, China; 2 Department of Cardiology, Guangzhou Women and Children’s Medical Center, Guangzhou Medical University, Guangzhou, China

**Keywords:** coronary vessels, humans, intravenous immunoglobulin, machine learning, SHAP algorithm

## Abstract

**Background:**

This study aimed to analyze the risk factors of coronary artery lesions (CAL) in patients with Kawasaki disease (KD) and establish predictive models for CAL in patients with KD.

**Methods:**

This retrospective cohort study included KD patients admitted to Shengjing Hospital of China Medical University, collecting data on 41 demographic, clinical, and laboratory parameters. LASSO regression identified key predictive variables. The dataset was split into 70% training and 30% validation. Ten models were trained using 10-fold cross-validation, with the training set balanced through ROSE oversampling. Model performance was assessed using the area under the receiver operating characteristic curve (AUC), sensitivity, specificity, and accuracy.

**Results:**

The CatBoost algorithm achieved the best results: AUC, 0.953; sensitivity, 0.908; specificity, 0.860; and accuracy, 0.883. Internal validation results were as follows: AUC, 0.874; sensitivity, 0.721; specificity, 0.848; accuracy, 0.837. External validation results were as follows: AUC, 0.876.sensitivity, 0.894; specificity, 0.954.

**Conclusions:**

We present a machine-learning model that predicts the risk of CAL in patients with KD in China, aiding doctors in creating personalized treatment strategies to improve outcomes.

## 1 Introduction

Kawasaki Disease (KD; mucocutaneous lymph node syndrome) is an immune-mediated acute systemic small- and medium-sized vasculitis that primarily affects children < 5 years of age. KD has been successively reported in over 60 countries and regions worldwide and is a leading cause of acquired pediatric heart disease (1). The most serious complication of KD is coronary artery lesions (CAL), which occur in 20–25% of untreated patients and lead to coronary artery thrombosis, stenosis, myocardial ischemia, and even sudden death. Although the incidence of CAL is reduced to approximately 4% with the use of intravenous immunoglobulin (IVIG), 10–20% of patients with KD do not respond to the first dose of IVIG and have a significantly higher risk of developing CAL (2–4).

Recently, risk factors for IVIG resistance and CAL have been extensively analyzed. A series of risk prediction models has been established, including those by Kobayashi et al. (5) in Japan, who first proposed a prediction scoring system for IVIG resistance in 2006; Son et al. (6), who established a prediction model for CAL based on the Z-score in North American centers in 2017; and Azuma et al. (7), who established a structural equation model and neural network analysis for predicting CAL through a single-center retrospective study in 2020. However, risk prediction models for CAL based on demographic characteristics, clinical manifestations, and laboratory test results differ significantly between races and regions (8). For example, models from the United States, Japan, and other regions are of limited value in predicting the occurrence of CAL in China, whereas prediction models in China apply to a single center but have relatively limited applicability to the general population. Therefore, we analyzed the risk factors of CAL and developed a risk prediction model for Northeast China. Rigorous external validation was conducted to make the prediction model applicable across China.

## 2 Methods

### 2.1 Patients and definitions

Patients clinically diagnosed with KD at Shengjing Hospital, China Medical University, from July 2012 to March 2024 were included in this retrospective study. Inclusion criteria: Patients diagnosed with Kawasaki disease according to the 2024 American Heart Association (AHA) guidelines (2); Age ≤ 18 years at the time of diagnosis; Complete medical records available for review. Exclusion criteria: The exclusion criteria encompassed the following cases (1): individuals with incomplete clinical data or those lost to follow-up (2); patients complicated by congenital heart disease (with the exception of mild shunt types, such as small atrial septal defects and patent ductus arteriosus) (3); those with primary immunodeficiency disorders (4); individuals suffering from autoimmune diseases (5); patients with malignant tumors or other severe chronic conditions. A total of 4421 children were included in this study. Additionally, patients with KD admitted to Guangzhou Women and Children’s Medical Center between January 2023 and December 2024 were enrolled as an external validation cohort.

All patients received IVIG (2 g/kg administered as a single intravenous infusion or 1 g/kg per day for 2 consecutive days), together with aspirin (30–50 mg/kg/day). Aspirin was reduced to a maintenance dose (3–5 mg/kg) after fever resolution and continued for 6–8 weeks. CAL or coronary artery aneurysm (CAA) was diagnosed using the absolute diameter and body surface area-corrected coronary Z-score (2) as follows: No involvement: Always <2; Dilation only: 2 to <2.5; or if initially <2, a decrease in Z score during follow-up ≥1; Small aneurysm: ≥2.5 to <5; Medium aneurysm: ≥5 to <10, and absolute dimension <8 mm; Large or giant aneurysm: ≥10, or absolute dimension ≥8 mm.

This study was approved by the Medical Ethics Committee of Shengjing Hospital of China Medical University (Approval Number: 2023PS776K). This study was approved by the Medical Ethics Committee of Guangzhou Women and Children’s Medical Center, Guangzhou Medical University (Approval Number:[2025]389B00).

### 2.2 Data collection

The following clinical information was obtained from the electronic medical records database at our hospital (1): general information (age [months], sex, season of onset) (2); main clinical manifestations (rash, cicatricial spots, conjunctival congestion, oral changes, enlarged cervical lymph nodes, sclerosis of the hands and feet in the acute phase, or periungual desquamation in the subacute phase), fever duration, initial treatment time of IVIG (3); laboratory findings before initial IVIG treatment, including routine blood count (white blood cell count [WBC], neutrophil percentage [NEUT], hemoglobin [HB], platelet count [PLT]), erythrocyte sedimentation rate [ESR], C-reactive protein (CRP), interleukin-6 (IL-6), N-terminal pro-brain natriuretic peptide (NT-proBNP), serum albumin (ALB), albumin-transferase (ALT), serum sodium ions (Na^+^), serum total bilirubin level (TBIL), and D-dimer (DD); if more than one value was present before the initial treatment, the maximum or minimum values were taken as appropriate (4); presence of infection, mixed infections, Pathogenic test results, including Mycoplasma pneumoniae, Chlamydia pneumoniae, Epstein-Barr virus, Streptococcus, herpes simplex virus, influenza virus, echovirus, Adenovirus, Coxsackievirus, Norovirus, Rotavirus (5); cardiac ultrasound was performed during hospitalization and outpatient follow-up (6). The definition of KD subtypes (complete KD/incomplete KD) and IVIG resistance are based on AHA guidelines.

### 2.3 Statistical analyses

#### 2.3.1 Sample size and missing data

As a retrospective study developing a predictive model from existing data, no formal *a priori* sample size calculation was performed. To ensure robustness, 10-fold cross-validation was employed. A *post-hoc* assessment using the Events Per Variable (EPV) criterion was conducted. With 370 coronary artery lesion (CAL) positive events and approximately 9 predictors in the final model, the EPV was 41, which meets common standards for predictive model development. Regarding missing data, cases with >5% missing data in any key variable were excluded. For the remaining dataset with minimal random missingness (≤5% per variable), multiple imputation was performed using the micepackage (version 3.16.0) in R to generate 5 imputed datasets, and results were pooled according to Rubin’s rules.

#### 2.3.2 Descriptive and modeling analysis

Statistical analyses were performed using R version 4.4.0 (R Core Team, Vienna, Austria). Categorical variables were analyzed using the χ² test; data are presented as frequencies (%). Non-normally distributed continuous variables were analyzed using the Mann–Whitney U test; data are summarized as medians and interquartile ranges. Statistical significance was set at P <0.05. Least Absolute Shrinkage and Selection Operator (LASSO) and multivariate logistic regression analyses were employed to identify independent risk factors for CAL. The dataset was divided into training (70%) and testing (30%). The ROSE method was applied for oversampling to address class imbalance. All machine-learning models were trained on the training set, including logistic regression, support vector machine (SVM), extreme gradient boosting (XGBoost), random forest, artificial neural network, gradient boosting machine (GBM), AdaBoost, LightGBM, CatBoost, and SVM with polynomial kernels. Receiver operating characteristic (ROC) curves were generated to assess the predictive performance of each model based on the area under the curve (AUC), sensitivity, specificity, accuracy, and 95% confidence interval (CI).

## 3 Results

### 3.1 Basic information

A total of 4,421 children participated in the coronary artery lesion risk factor analysis (**Table 1**), including 2,739 boys and 1,682 girls, with a male-to-female ratio of 1.63:1. In line with recent epidemiological surveys, the male-to-female ratio of KD was 1.5–1.7:1 (1, 9). The median age at disease onset was 25 months (interquartile range, 14–39 months). Of the participants, 4,051 (91.6%) were classified as coronary artery lesion-negative (without coronary artery abnormalities), while 370 (8.4%) developed CAL, indicating positive coronary artery involvement. The incidence of CAL in KD patients was 8.4%.

**Table 1:** Baseline table of clinical indicators for coronary artery lesion-negative and coronary artery lesions.

Variables	Total (n = 4421)	Coronary artery lesion-negative 0 (n = 4051)	Coronary artery lesions 1 (n = 370)	P value
Sex, n (%)				< 0.001
Female	1682 (38)	1584 (39)	98 (26)	
Male	2739 (62)	2467 (61)	272 (74)	
Immunoglobulin, n (%)				< 0.001
Heat Inactivation	2528 (57)	2361 (58)	167 (45)	
Physical Filtration	1893 (43)	1690 (42)	203 (55)	
Season, n (%)				0.387
Spring	990 (22)	904 (22)	86 (23)	
Summer	1234 (28)	1145 (28)	89 (24)	
Autumn	1176 (27)	1071 (26)	105 (28)	
Winter	1021 (23)	931 (23)	90 (24)	
Types of KD, n (%)				0.116
Classic KD	3447 (78)	3171 (78)	276 (75)	
Incomplete KD	974 (22)	880 (22)	94 (25)	
IVIG reaction, n (%)				< 0.001
IVIG-sensitive	3998 (90)	3702 (91)	296 (80)	
IVIG-resistance	423 (10)	349 (9)	74 (20)	
Infection, n (%)	2030 (46)	1910 (47)	120 (32)	< 0.001
Mix infection, n (%)	824 (19)	777 (19)	47 (13)	0.003
MP, n (%)	1263 (29)	1190 (29)	73 (20)	< 0.001
Streptococcus, n (%)	95 (2)	89 (2)	6 (2)	0.587
EBV, n (%)	193 (4)	182 (4)	11 (3)	0.216
HSV, n (%)	251 (6)	235 (6)	16 (4)	0.29
RV, n (%)	132 (3)	127 (3)	5 (1)	0.077
NV, n (%)	77 (2)	72 (2)	5 (1)	0.695
ADV, n (%)	91 (2)	85 (2)	6 (2)	0.669
Influenza virus, n (%)	521 (12)	491 (12)	30 (8)	0.027
CP, n (%)	144 (3)	136 (3)	8 (2)	0.277
CV, n (%)	120 (3)	118 (3)	2 (1)	0.012
ECHO, n (%)	74 (2)	72 (2)	2 (1)	0.118
Rash	3396 (77)	3127 (77)	269 (73)	0.058
BCG	715 (16)	656 (16)	59 (16)	0.96
Con	3802 (86)	3473 (86)	329 (89)	0.107
Lips and oral	3835 (87)	3513 (87)	322 (87)	0.931
Hand and foot	2337 (53)	2146 (53)	191 (52)	0.657
Cer	3971 (90)	3634 (90)	337 (91)	0.455
Per	1727 (39)	1565 (39)	162 (44)	0.059
Age	25 (14, 39)	26 (14, 43)	20.5 (9.25, 35)	< 0.001
Initial IVIG days	6 (5, 8)	6 (5, 7)	7 (5.25, 9)	< 0.001
Days of fever	7 (6, 8)	7 (6, 8)	8 (6, 11)	< 0.001
WBC (*10^9/L)	13.1 (10.04, 16.9)	13.06 (10, 16.8)	14.5 (10.7, 18.2)	< 0.001
NE(%)	64.1 (51.6, 75.6)	64.1 (51.5, 75.3)	64.25 (52.82, 76.8)	0.664
HB(g/L)	110 (103, 117)	110 (103, 117)	107 (99, 114)	< 0.001
PLT(*10^9/L)	360 (281, 459)	358 (281, 456)	378.5 (278, 485.75)	0.065
ESR (mm/h)	61 (44, 79)	60 (43, 79)	62 (45, 80)	0.244
CRP (mg/L)	41.1 (16.8, 78.24)	37.6 (15.8, 75.1)	89.5 (51.77, 127)	< 0.001
IL-6 (ng/L)	305 (68.11, 311)	310 (68.63, 321)	275 (66.56, 278)	< 0.001
BNP (pg/mL)	421 (154.3, 1151)	404 (150.9, 1128.5)	586.65 (215.2, 1641.25)	< 0.001
ALB(g/L)	34.7 (31.7, 37.7)	35.1 (32.2, 38)	29.6 (26.8, 33.3)	< 0.001
ALT (U/L)	21 (12, 52)	21 (12, 52)	24 (13, 50)	0.24
TBIL(umol/L)	4.8 (3.4, 7.4)	4.9 (3.4, 7.4)	4.56 (3.13, 7.9)	0.359
Na+(mmol/L)	136 (134, 138)	136 (134, 138)	136 (134, 138)	0.02
D-D(ug/L)	431 (259, 742)	423 (256, 728)	537 (297, 924.25)	< 0.001

### 3.2 Correlation analysis of risk factors for CAL in KD​

**Figure 1:**
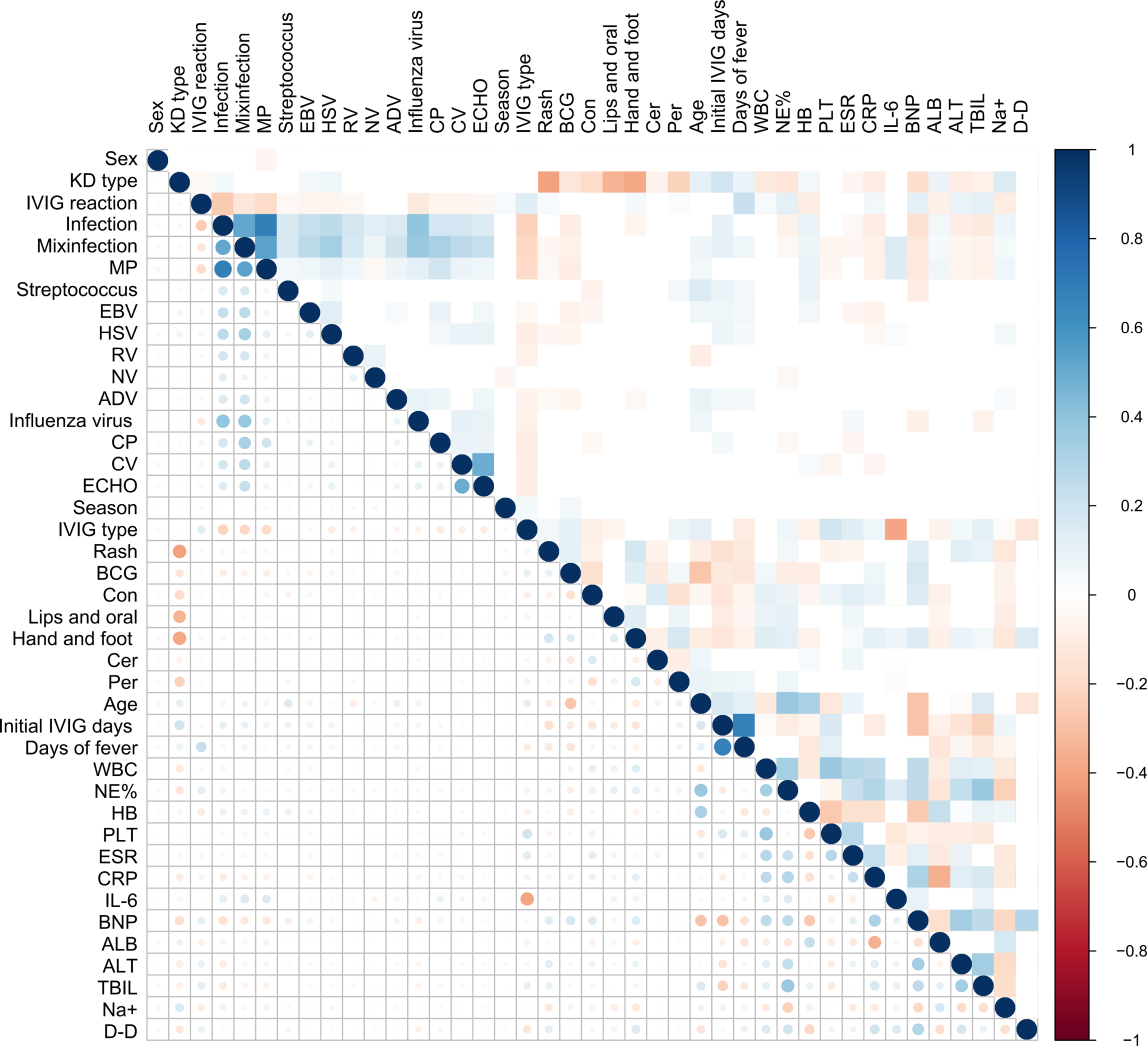
Correlation heatmap analysis between various clinical factors and coronary artery outcomes in KD.

The correlation heatmap (**Figure 1**) revealed distinct associations between clinical variables and treatment outcomes in Kawasaki disease. IVIG resistance showed positive correlations with the presence of infection (particularly mixed infection and Mycoplasma pneumoniae), longer duration of fever, delayed initiation of IVIG therapy, and elevated inflammatory markers, including CRP, BNP, and D−dimer. Conversely, serum albumin and hemoglobin levels exhibited inverse correlations with these inflammatory indicators. Most individual pathogens, such as Streptococcus, EBV, HSV, and rotavirus, demonstrated only minimal correlation with IVIG response or disease subtype. Furthermore, clinical signs, including rash and mucosal changes, displayed no strong linear association with laboratory parameters or treatment outcomes.

### 3.3 Feature selection

**Figure 2:**
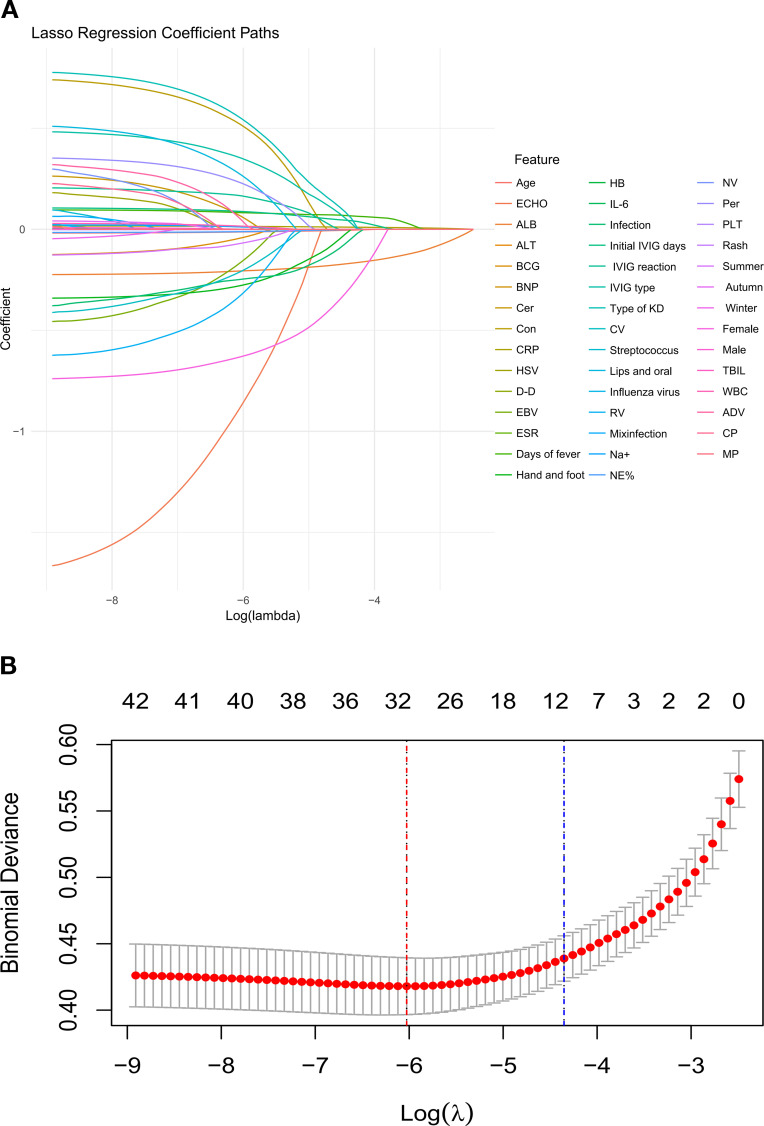
LASSO regression analysis for feature selection in Kawasaki disease. **(A)** The LASSO coefficient paths for 41 features. **(B)** Selected parameter λ that minimizes the mean squared error, identifying 10 main predictive features for the model.

Lasso regression employs L1 regularization (λ penalty term) to shrink the coefficients of less influential variables to zero. This process effectively selects the features with the strongest predictive power for the target variable, thereby simplifying the model and enhancing its generalization capability. We chose the core variables corresponding to lambda.lse (λ = 0.01287), i.e., the penalty parameter that minimizes the mean square error (or binomial deviation) of the model during cross-validation, and the variables screened by this parameter will be used for subsequent model construction. The results showed the LASSO coefficient profiles for the 41 features (**Figure 2A**), demonstrating the dynamic process of LASSO screening variables. A total of 41 variables from the training set were included in the LASSO regression analysis, utilizing ten-fold cross-validation for model selection (**Figure 2B**). The contraction parameter λ of 0.01287 was selected based on the cross-validation results, yielding 10 potential predictors, including sex, immunoglobulin inactivation process (physical filtration vs. heat inactivation), age, timing of initial IVIG administration, presence of infections, duration of fever, and laboratory parameters before the initial IVIG dose: hematologic indicators (neutrophil percentage), inflammatory markers (CRP), serum albumin, alanine aminotransferase, all of which have been linked to coronary artery aneurysm development.

### 3.4 Univariate and multivariate logistic analyses of KD presenting with coronary artery aneurysms

To further screen relevant variables and exclude redundant terms, we performed univariate and multivariate logistic regression analyses of the data based on the variables identified by the LASSO regression. **Table 2** presents the results of univariate logistic regression analysis, which identified significant associations between various factors and the development of coronary artery aneurysms. These factors included male sex, physical filtration of IVIG, presence of concurrent infections, age, timing of initial IVIG administration, fever duration, serum albumin, and C-reactive protein and incomplete KD. All these factors were significantly correlated with coronary artery aneurysm formation (P<0.05). Upon further validation in multivariate analysis, male sex (OR 2.22, 95%CI 1.59–3.11), incomplete Kawasaki disease (OR 1.83, 95%CI 1.29–2.62), physical filtration IVIG method (OR 1.44, 95%CI 1.06–1.97), concurrent infection (OR 0.70, 95%CI 0.51–0.96), younger age (OR 0.98, 95%CI 0.98–0.99), delayed initial IVIG administration (OR 1.09, 95%CI 1.02–1.17), prolonged duration of fever (OR 1.09, 95%CI 1.03–1.17), elevated C-reactive protein (OR 1.01, 95%CI 1.01–1.02), and decreased serum albumin (OR 0.81, 95%CI 0.78–0.84) were identified as independent factors associated with coronary artery outcomes. Neutrophil percentage (NE%) was not retained in the final multivariate model. Based on the findings of both the univariate and multivariate regression analyses, we selected variables with significant associations (P<0.05) in the multivariate logistic analysis for incorporation into the machine-learning models. This approach aims to enhance the prediction and interpretation of coronary artery aneurysm development in patients with KD.

**Table 2:** The result of univariate and multivariate logistic regression and analysis for training set.

Characteristics	Category	Univariate logistic analysis		Multivariate logistic analysis	
		OR (95CI%)	P value	OR (95CI%)	P value
Sex	Female	Ref	Ref	Ref	Ref
	Male	2.15 (1.60-2.89)	<0.001	2.22 (1.59-3.11)	<0.001
Types of KD	Classic KD	Ref	Ref	Ref	Ref
	Incomplete KD	1.34 (1.00-1.79)	0.048	1.83 (1.29-2.62)	<0.001
Infection	Yes	0.54 (0.41-0.70)	<0.001	0.70 (0.51-0.96)	0.029
Immunoglobulin	Heat Inactivation	Ref	Ref	Ref	Ref
	Physical Filtration	1.78 (1.38-2.30)	<0.001	1.44 (1.06-1.97)	0.02
Age	\	0.99 (0.98-0.99)	<0.001	0.98 (0.98-0.99)	<0.001
Initial IVIG days	\	1.13 (1.09-1.18)	<0.001	1.09 (1.02-1.17)	0.014
Days of fever	\	1.16 (1.12-1.21)	<0.001	1.09 (1.03-1.17)	0.007
NE(%)	\	1.00 (1.00-1.01)	0.461	\	\
CRP (mg/L)	\	1.02 (1.02-1.02)	<0.001	1.01 (1.01-1.02)	<0.001
ALB(g/L)	\	0.76 (0.73-0.79)	<0.001	0.81 (0.78-0.84)	<0.001

### 3.5 Model performance comparison

**Figure 3:**
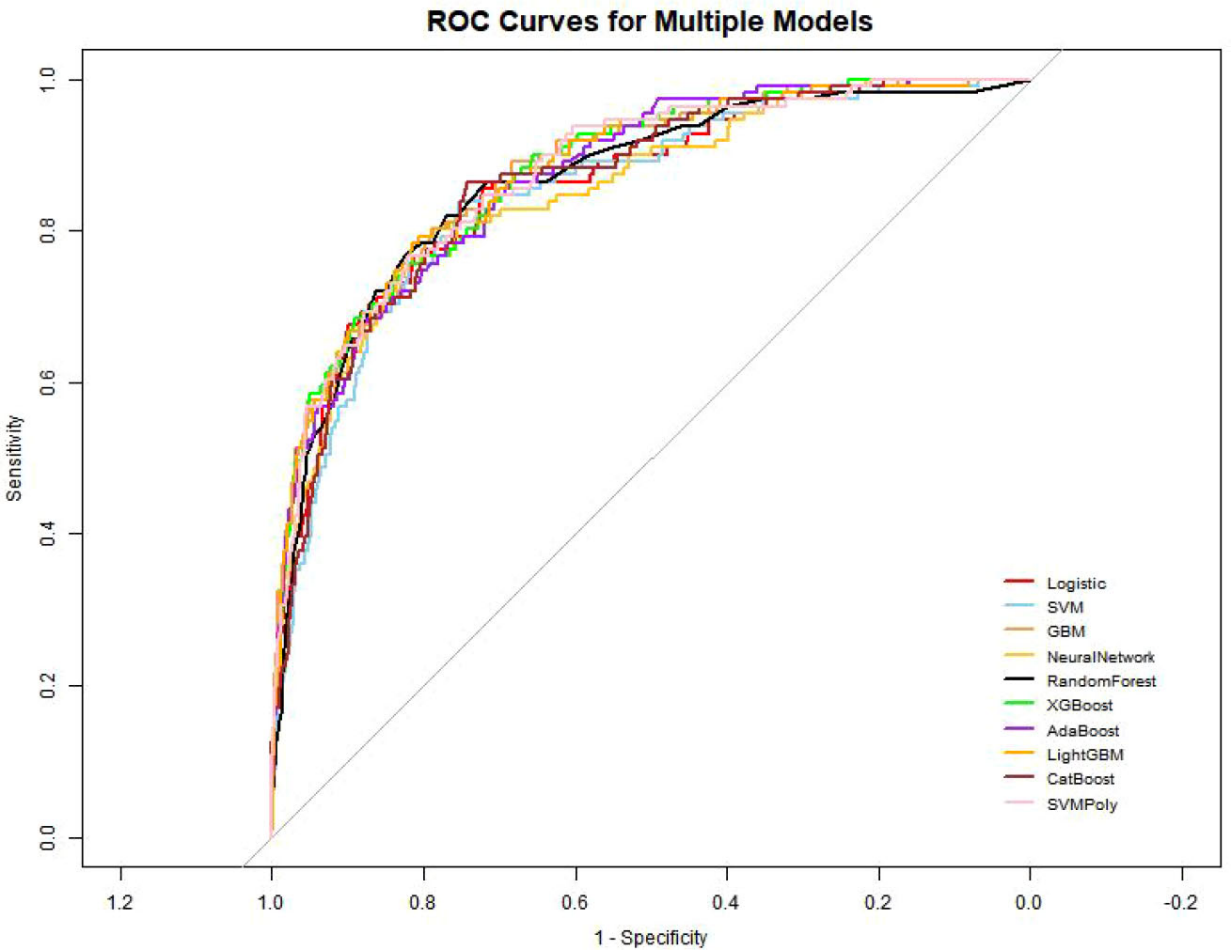
ROC curves for machine learning models predicting CAL in KD.

Based on the performance evaluations across both the training and test sets, the CatBoost model was selected as the optimal predictive model for artery aneurysms in this study. We trained ten models—logistic regression, SVM, GBM, neural network, random forest, XGBoost, AdaBoost, LightGBM, CatBoost, and polynomial kernel SVM—using 10-fold cross-validation and balanced the training set data with up-sampling techniques. The performance of each model on both the training and test sets was evaluated using the AUC, sensitivity, specificity, and accuracy metrics (**Table 3**). **Figure 3** presents the corresponding ROC curves.

**Table 3:** Performance comparison of ten machine learning (ML) models.

Data Set	Model	AUC	95%CI	Sensitivity	Specificity	Accuracy
Training set	Logistic	0.860	0.850-0.869	0.772	0.779	0.775
	SVM	0.895	0.886-0.903	0.817	0.802	0.810
	GBM	0.926	0.919-0.932	0.881	0.84	0.861
	NeuralNetwork	0.874	0.866-0.884	0.782	0.796	0.789
	RandomForest	0.885	0.876-0.893	0.784	0.821	0.803
	XGBoost	0.942	0.937-0.948	0.878	0.847	0.862
	AdaBoost	0.928	0.921-0.934	0.849	0.833	0.841
	LightGBM	0.937	0.931-0.944	0.893	0.831	0.862
	CatBoost	0.953	0.948-0.958	0.908	0.860	0.883
	SVMPoly	0.883	0.875-0.892	0.805	0.779	0.792
Testing set	Logistic	0.858	0.820-0.896	0.774	0.772	0.772
	SVM	0.849	0.811-0.887	0.766	0.787	0.785
	GBM	0.878	0.844-0.911	0.748	0.824	0.818
	NeuralNetwork	0.864	0.812-0.890	0.766	0.790	0.788
	RandomForest	0.868	0.826-0.901	0.764	0.824	0.820
	XGBoost	0.877	0.844-0.910	0.712	0.841	0.830
	AdaBoost	0.870	0.837-0.904	0.711	0.831	0.821
	LightGBM	0.875	0.841-0.910	0.748	0.834	0.827
	CatBoost	0.874	0.840-0.908	0.721	0.848	0.837
	SVMPoly	0.862	0.827-0.897	0.784	0.774	0.775

**Figure 4:**
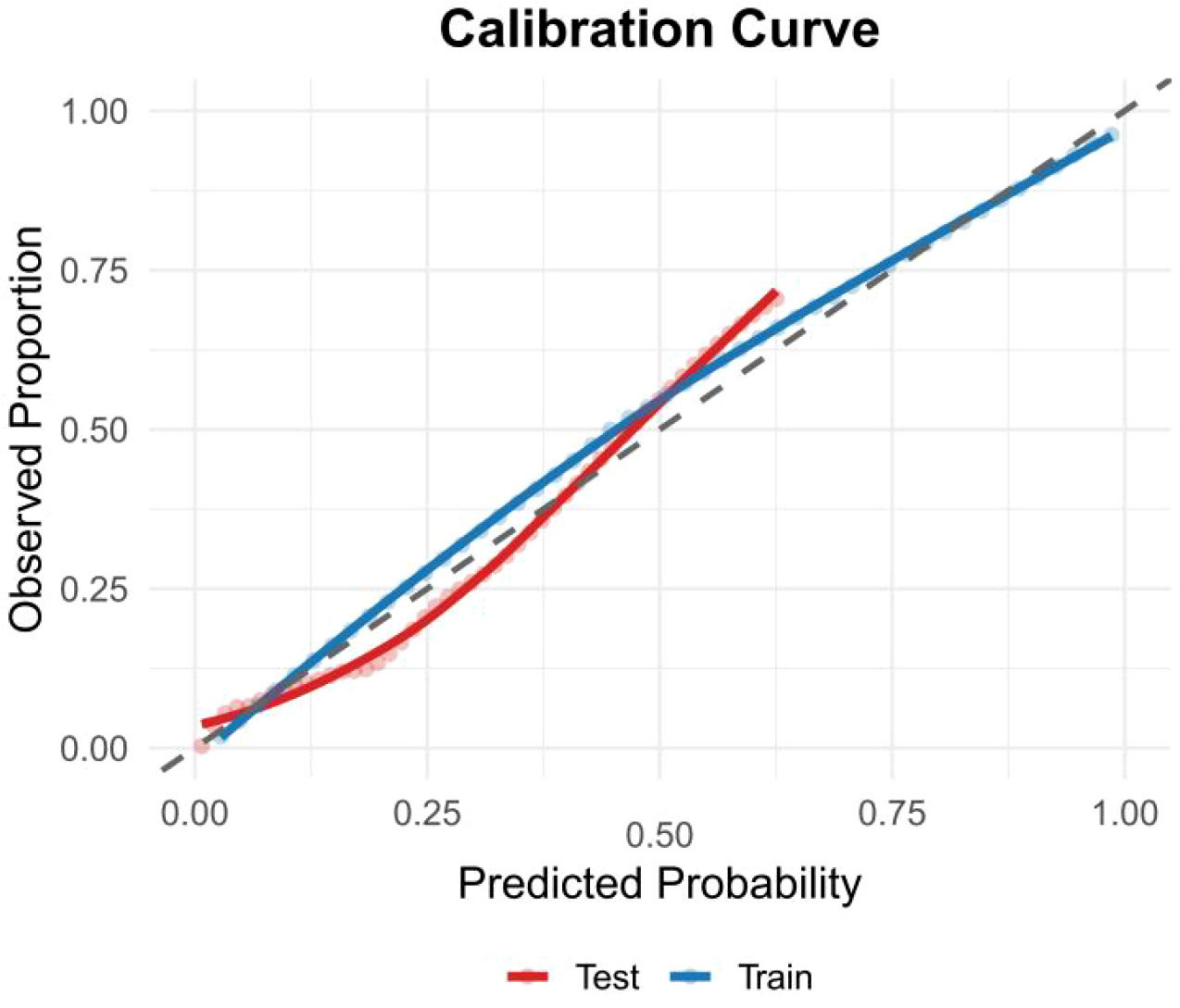
The calibration curve reflects the consistency between the training set and the test set.

In the training set, CatBoost achieved the highest AUC (0.953), followed by XGBoost (0.942) and LightGBM (0.937). With a sensitivity of 0.908, a specificity of 0.860, an accuracy of 0.883, and the highest AUC, CatBoost demonstrated strong generalization capability. In the test set, CatBoost attained an AUC of 0.874 (95% confidence interval: 0.840–0.908), confirming its robustness on unseen data. It also exhibited high specificity (0.848), moderate sensitivity (0.721), and high accuracy (0.837).The calibration curve also reflects the consistency between the training set and the test set (**Figure 4**). This balanced and consistent performance across both datasets established CatBoost as the preferred model for predicting the risk of coronary artery aneurysm in patients with Kawasaki disease.

### 3.6 External validation analysis of the CatBoost model for coronary artery aneurysm prediction in KD

**Figure 5:**
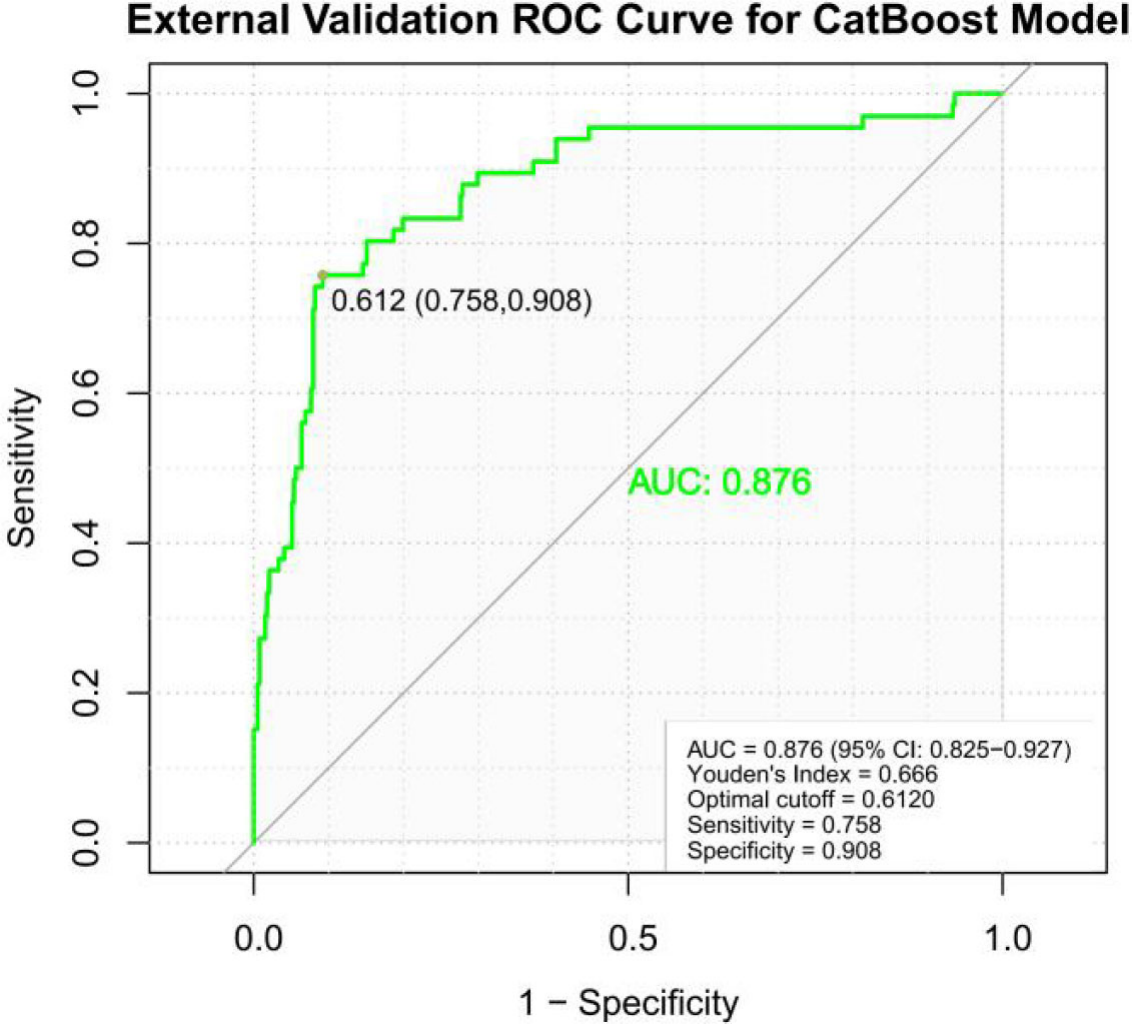
External validation.

An external validation cohort comprising 457 patients with KD admitted to the Guangzhou Women and Children’s Medical Center between January 2023 and December 2024 was used to evaluate the generalizability and clinical applicability of the previously developed CatBoost prediction model for coronary artery aneurysm development. The validation results (**Figure 5**) demonstrated excellent performance across all metrics; the model achieved a sensitivity of 0.758, specificity of 0.908, indicating a strong discriminatory ability to identify both positive and negative cases. Most notably, the model attained an AUC of 0.876 (95% confidence interval: 0.825-0.927) on the external validation set, confirming its robust predictive performance and generalizability across different patient populations and temporal settings. These results indicate that the CatBoost model maintains high diagnostic accuracy and reliability when applied to new clinical data from an independent medical center, supporting its potential utility as a clinical decision support tool for the early identification of patients with KD at high risk of developing coronary artery complications.

### 3.7 Interpretation of the CatBoost model based on SHAP analysis

**Figure 6:**
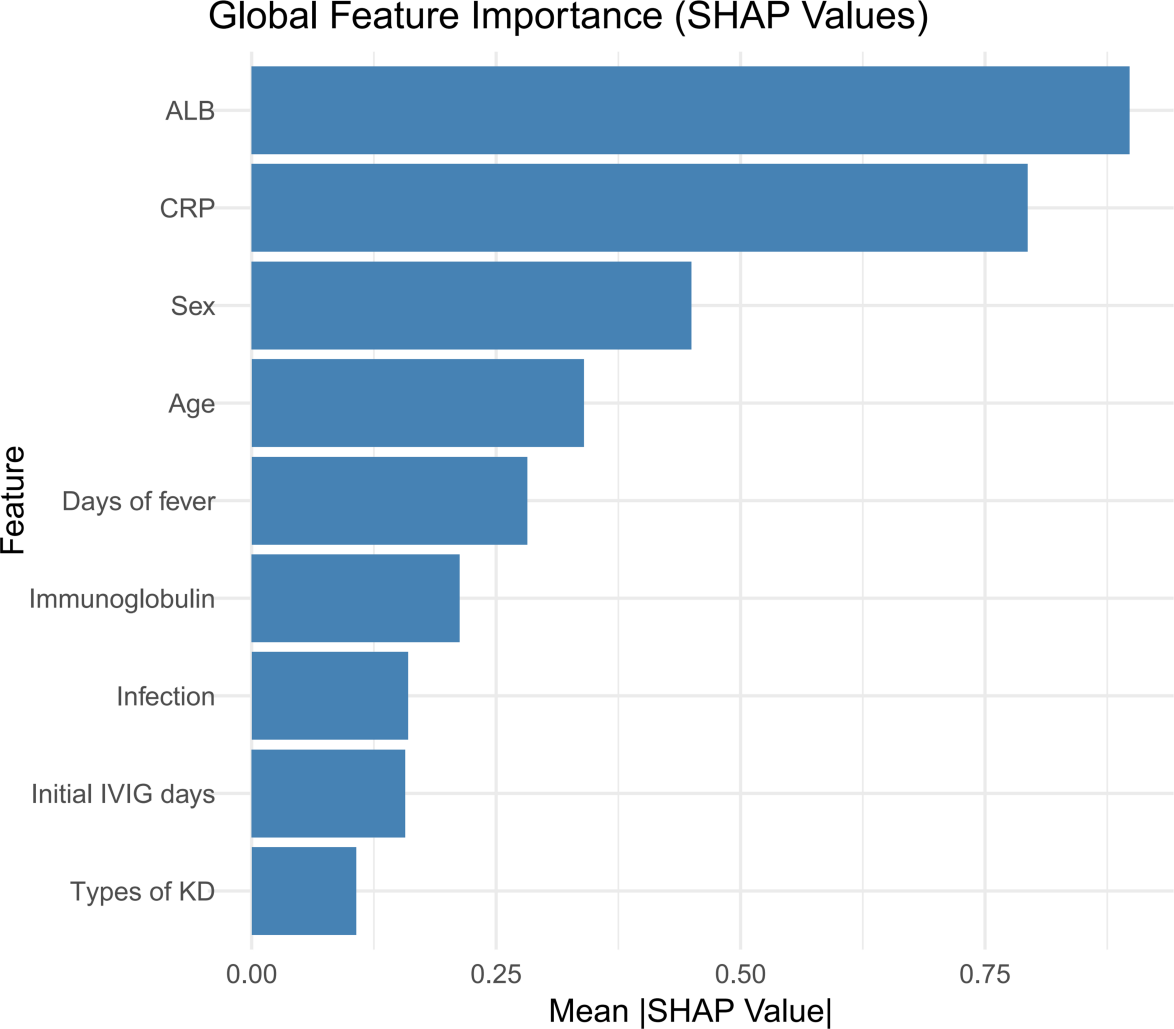
SHAP feature importance analysis of the CatBoost model.

**Figure 7:**
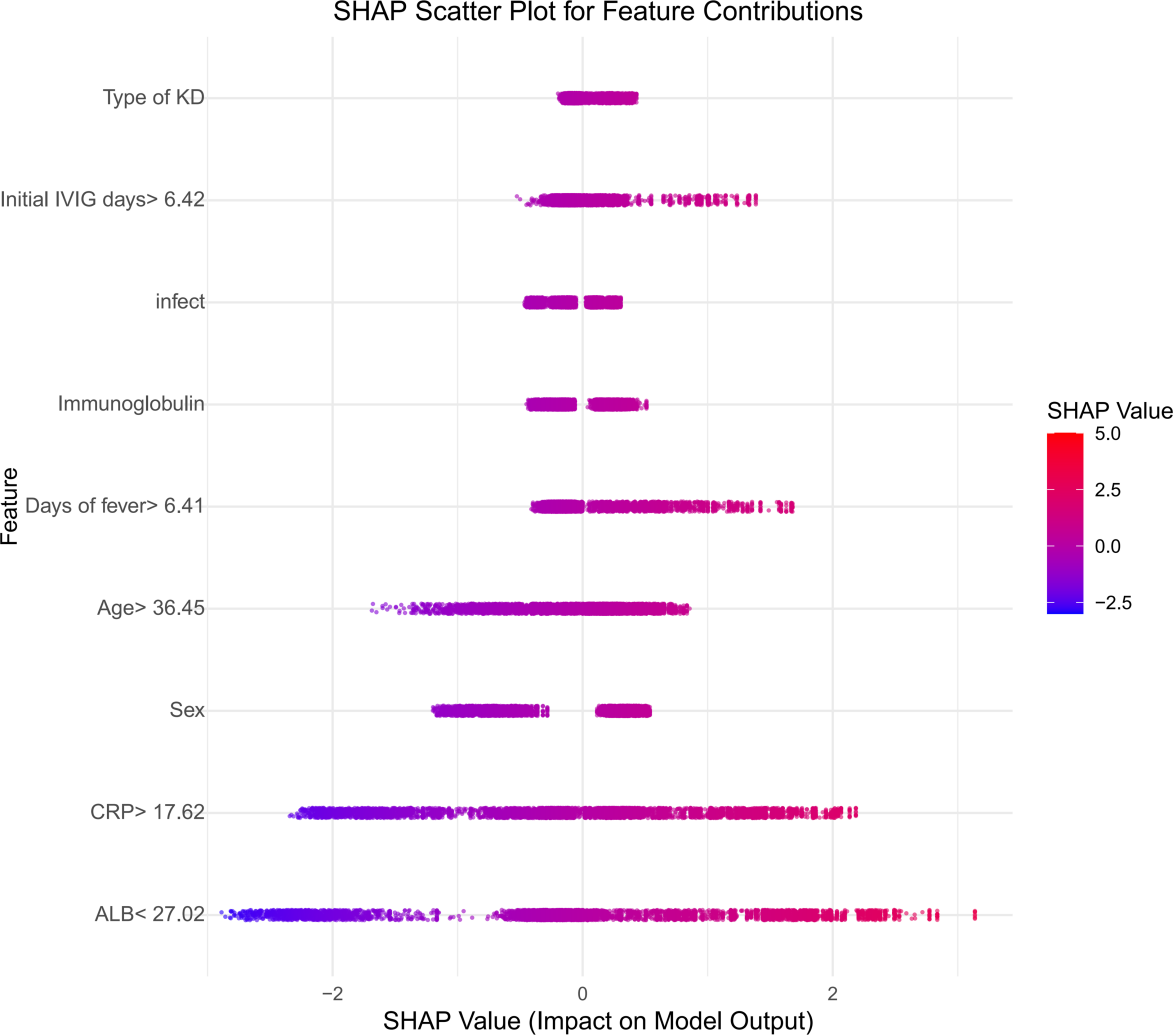
SHAP summary plot illustrating feature effects on CAL prediction.

SHAP analysis, which is based on the Shapley value theory from game theory, is a technique used to interpret the machine-learning model outcomes. This method provides a clear framework for assessing the significance of individual features by quantifying their contributions to model predictions. It not only identifies the influence of each feature on the results, but also offers a global interpretation that helps explain the model’s behavior. In the CatBoost model for predicting artery aneurysms in patients with KD, the SHAP feature importance ranking demonstrated the relative contributions of various clinical features (**Figure 6**).The global feature importance plot revealed that serum ALB, CRP, and male sex were the most influential predictors, followed by age at onset, duration of fever. Additionally, the SHAP contribution plot (**Figure 7**) illustrates how these features affect individual risk predictions: elevated CRP levels, male sex, types of KD and prolonged fever duration are associated with an increased risk of coronary artery aneurysm formation (represented by red dots), whereas higher albumin levels, presence of concurrent infections, and earlier IVIG administration are protective factors (represented by blue dots). The analysis indicated that specific IVIG preparation methods (particularly physical filtration) and a younger age at disease onset were significantly associated with an increased risk of coronary artery complications.

The model further established optimal predictive cut-off values for key continuous variables: age >36.45 months, initial IVIG delay >6.42 days, fever duration >6.41 days, CRP >17.62 mg/L, and albumin <27.02 g/L. These thresholds provide actionable quantitative criteria for clinical risk stratification. The calibration curve indicated good agreement between the training and test sets, supporting the model’s robust generalizability.

### 3.8 Analysis of the interpretation of single-sample predictive features

**Figure 8:**
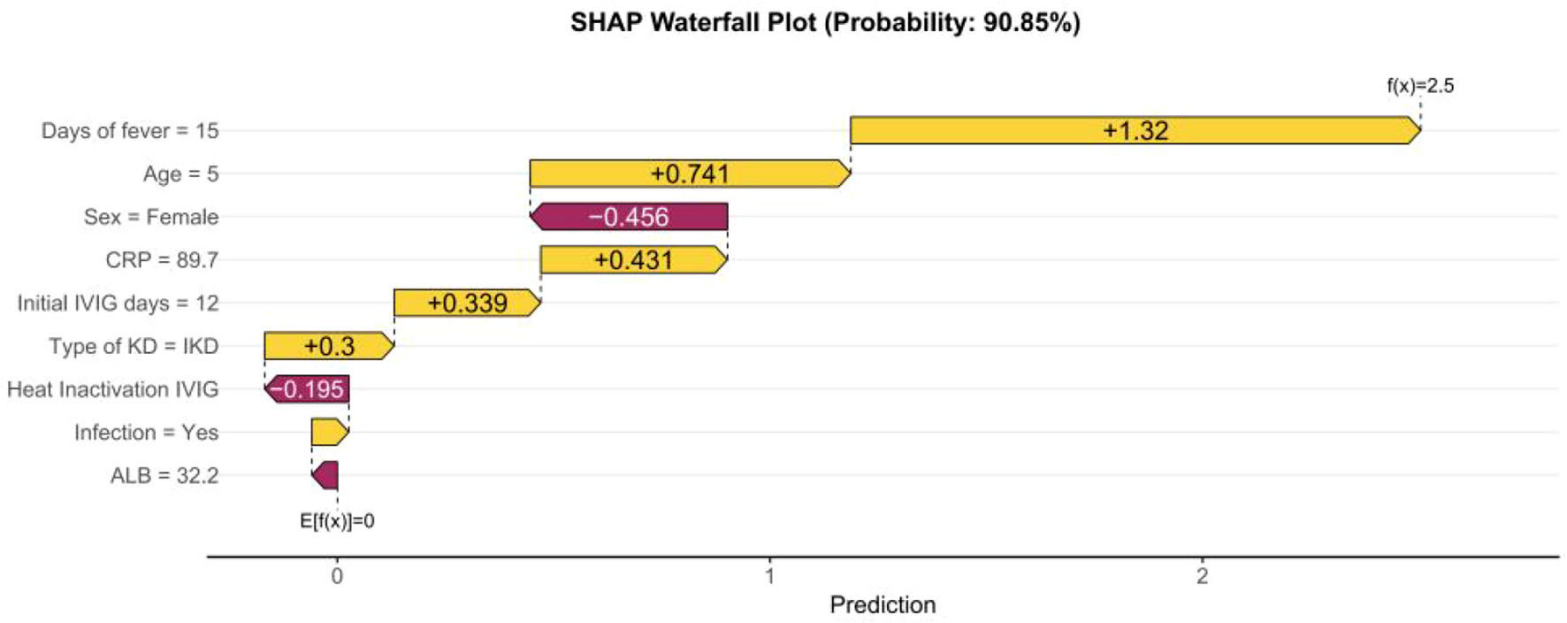
Characteristic contribution plot of a patient with KD.

The SHAP waterfall plot (**Figure 8**) for a patient with a 15-day fever duration, age of 5 months, female sex, CRP level of 89.7 mg/L, 12 days of initial IVIG delay, incomplete KD (IKD) type, heat-inactivated IVIG preparation, concurrent infection, and serum albumin level of 32.2 g/L clearly demonstrates the drivers of coronary artery aneurysm prediction for this individual. A 15-day fever duration was the primary contributor to the high-risk prediction, imparting the largest positive impact (+1.32). Younger age (5 months) positively contributed (+0.741), further elevating the predicted value. The CRP level (89.7 mg/L) added a positive contribution of +0.431, while the 12-day delay in initial IVIG administration contributed +0.339, and the IKD type contributed +0.3—all of which augmented the predicted probability. Being female (sex = female) acted as a notable protective factor, exerting a substantial negative impact (-0.456) that reduced the predicted value. Heat-inactivated IVIG preparation, concurrent infection, and lower serum albumin (32.2 g/L) contributed modestly to risk reduction with negative impacts of -0.195, a small negative contribution (visually ~-0.05), and -0.03, respectively. The cumulative positive and negative contributions of all features ultimately increased the predicted value from the baseline (E[f(X)] = 0) to 2.5, corresponding to a 90.85% probability of coronary artery aneurysm development. This indicates the model identified multiple significant risk factors (e.g., prolonged fever, young age, elevated CRP, IVIG delay, IKD type) alongside minor protective influences (e.g., female sex, heat-inactivated IVIG, infection, low albumin) for this patient’s KD-associated coronary complication risk.

## 4 Discussion

Machine-learning algorithms are widely utilized in the diagnosis and prognosis of cardiovascular and cerebrovascular disorders, diabetes, and other conditions (10–12). To our knowledge, this study represents one of the largest single-center cohorts used to develop a machine learning model for predicting CAL in KD. It is also the first to perform cross-regional external validation between Northeast and South China, suggesting the model may generalize across geographically distinct populations. The CatBoost model, based on a large dataset (n = 4,421) of patients in Northeast China, showed the best performance among the machine-learning algorithms. Internal validation of the model using 10-fold cross-validation confirmed its excellent performance and stability, and the external validation results reflected the model’s generalizability and robustness, making the prediction model applicable to the entire country.

To further understand the fundamental aspects of CAL, we used the SHAP algorithm to analyze the CatBoost model, which identified nine features with a considerable impact on CAL: serum ALB, CRP, sex, fever duration, age, Types of KD, initial treatment time of IVIG, IVIG inactivation processes, and infection. The top three variables influencing the model predictions were the CRP level, serum ALB level, and sex. Among these, the prediction of CAL in KD by IVIG inactivation was first reported.

Age, sex, incomplete KD, initial treatment time with IVIG, CRP, and ALB were similar to those in previous studies (4, 5, 13, 14). Age has been mentioned in many previous studies; the cut-off value is mostly 6 months or 1 year (10–12, 15–18). Although the optimal cutoff values selected in each study were different, age was considered to be a more dangerous risk factor. A total of 4,421 children participated in the coronary artery lesion risk factor analysis, including 2,739 boys and 1,682 girls, with a male-to-female ratio of 1.63:1. In line with recent epidemiological surveys, the male-to-female ratio of KD was 1.5–1.7:1 (1, 9). Among the 41 risk factors, When IVIG resistance was included in the full variable set for LASSO-based feature selection, its coefficient was compressed to zero, leading to its exclusion. This indicates that, IVIG resistance did not provide additional independent predictive information. This did not align with previous literature reports (5, 19, 20) indicating that IVIG resistance constitutes an independent risk factor for CAL in Kawasaki disease. There are three potential causes contributing to this outcome. Firstly, Information overlap and collinearity: IVIG resistance essentially reflects poor response to initial anti-inflammatory therapy and is strongly associated with many baseline clinical and laboratory indicators. Kids who are resistant to IVIG usually have stronger inflammatory responses (such higher CRP and neutrophil counts and decreased albumin) and longer fevers (14, 21, 22). Consequently, its predictive influence is probably encompassed by the directly measured inflammatory burden factors (e.g., CRP, albumin) and treatment timing variables (e.g., day of IVIG beginning) that persisted in the model. The LASSO regularization technique kept these direct measurements because they were more useful for keeping the model simple, but it got rid of the composite indicator “IVIG resistance.” Secondly, Quality of data and missing data: Even though we tried hard to get the information, the recording of IVIG resistance status in old medical records was not very complete. In several cases, there wasn’t enough documentation of ongoing temperature monitoring, which made it hard to tell if the fever really went away. About 15% of the IVIG resistance variable was missing, and there may have been bias in the recorded instances because the documentation wasn’t uniform. These problems with data quality could have made it less stable and less useful as a predictor. Thirdly, Limitations of the sample size: IVIG resistance happens in about 10–20% of all KD patients (23–25), which is about 423 cases in our training group. Even while the number is not modest in absolute terms, it may not have been enough to compete with continuous variables during regularization in a high-dimensional multivariate analysis.

This is the first study to explore the relationship between IVIG inactivation and CAL. Our findings show that CAL in KD patients are significantly linked to IVIG inactivation, which alters IgG molecule integrity and subsequently impacts inflammatory responses and therapeutic efficacy. Heat-inactivated IVIG, using pasteurization at 60–70 °C, disrupts IgG structural integrity. While this reduces IgG activity, it appears to enhance anti-inflammatory effects compared to physically inactivated IVIG, which uses 35 nm nanofiltration to preserve IgG activity and structure but may result in reduced efficacy. Previous studies (26) have shown that IVIG removes autoantibodies by binding to the Fc receptor, whereas heat inactivation may produce immunomodulatory fragments (e.g., Fc fragments) or oxidized products that potentiate the anti-inflammatory or immunomodulatory effects, thereby reducing the risk of IVIG resistance. In contrast, nanofiltration removes large molecular viruses, but retains fewer small molecules with immunomodulatory roles, potentially weakening IVIG’s immunoregulatory capacity. Moreover, heat inactivation may reduce the levels of the immunostimulatory components, further mitigating IVIG resistance in certain patient populations, indicating that heat-inactivated IVIG may reduce IVIG resistance through unique by-products or mechanisms, but physically inactivated IVIG, which is closer to its natural form, lacks these effects. However, further mechanistic studies and large-scale clinical trials are required to confirm these observations. Future research should focus on the distinct components of IVIG and their roles in CAL in patients with KD, particularly the contributions of different immunoactive components, to provide novel insights into the optimization of KD treatment.

With the continuous development of the information age, big data and artificial intelligence in medicine have become prominent. Machine learning, particularly adept at handling extensive data, is increasingly being harnessed in the diagnostic testing of KD to distinguish KD from other febrile diseases to avoid the risk of delayed diagnosis of CAL and has a higher predictive performance than regression algorithms (7, 24, 27–30).

This study had several limitations. First, this was a retrospective study, which has obvious limitations compared with prospective studies. The external validation cohort was limited in size and covered a relatively short time period. This restricted our ability to perform robust subgroup analyses and assess model stability across different clinical eras. A single external validation also provides limited evidence for generalizability. Additionally, the sample sizes for moderate-to-severe CAL cases and for certain pathogens (e.g., EBV, adenovirus) were too small to support reliable subgroup or multiclass prediction analyses. Our study focused only on acute-phase CAL and did not capture long-term outcomes such as aneurysm regression, myocardial ischemia, or cardiovascular events, limiting the model’s applicability for long-term follow-up.

In conclusion, this retrospective cross-sectional study analyzed the risk factors for CAL in Chinese children with KD were analyzed and constructed a robust prediction model, CatBoost, by using SHAP analysis. We also performed external validation to demonstrate the strong generalization ability of the model, making it applicable to China. We revealed, for the first time, a relationship between IVIG inactivation and CAL: CAL in patients with KD are significantly associated with IVIG inactivation. In future studies, we will strive for a multi-center prospective study, and further adjust the machine-learning algorithm to improve the predictive value of the model.

## Data Availability

The raw data supporting the conclusions of this article will be made available by the authors, without undue reservation.
